# Canonical notch activation in patients with scrub typhus: association with organ dysfunction and poor outcome

**DOI:** 10.1007/s15010-024-02192-2

**Published:** 2024-03-19

**Authors:** Jan K. Damås, Kari Otterdal, Elisabeth Astrup, Tove Lekva, Jeshina Janardhanan, Annika Michelsen, Pål Aukrust, George M. Varghese, Thor Ueland

**Affiliations:** 1 Research Institute of Internal Medicine, Oslo University Hospital Rikshospitalet, Oslo, Norway; 2 Section of Clinical Immunology and Infectious Diseases, Oslo University Hospital Rikshospitalet, Oslo, Norway; 3 Faculty of Medicine, University of Oslo, Oslo, Norway; 4 Institute of Clinical Medicine, Akershus University Hospital, Lørenskog, Norway; 5 Department of Clinical and Molecular Medicine, Norwegian University of Science and Technology, Trondheim, Norway; 6 Department of Infectious Diseases, St. Olavs Hospital, Trondheim, Norway; 7 Thrombosis Research Center (TREC), Division of Internal Medicine, University Hospital of North Norway, Tromsø, Norway; 8 Department of Medicine, Christian Medical College, Vellore, Tamil Nadu India; 9 Department of Infectious Diseases, Christian Medical College, Vellore, Tamil Nadu India

**Keywords:** Scrub typhus, *Orientia tsutsugamushi*, Notch signaling, Inflammation, Delta-like 1

## Abstract

**Purpose:**

The mechanisms that control inflammation in scrub typhus are not fully elucidated. The Notch pathways are important regulators of inflammation and infection, but have not been investigated in scrub typhus.

**Methods:**

Plasma levels of the canonical Notch ligand Delta-like protein 1 (DLL1) were measured by enzyme immunoassay and RNA expression of the Notch receptors (*NOTCH1*, *NOTCH2* and *NOTCH4*) in whole blood was analyzed by real-time PCR in patients with scrub typhus (*n* = 129), in patients with similar febrile illness without *O. tsutsugamushi* infection (*n* = 31) and in healthy controls (*n* = 31); all from the same area of South India.

**Results:**

Our main results were: (i) plasma DLL1 was markedly increased in scrub typhus patients at hospital admission with a significant decrease during recovery. (ii) RNA expression of *NOTCH4* was decreased at admission in whole blood. (iii) A similar pattern for DLL1 and *NOTCH4* was seen in febrile disease controls. (iv) Admission DLL1 in plasma was associated with disease severity and short-term survival. (vi) Regulation of Notch pathways in *O. tsutsugamushi-*infected monocytes as evaluated by public repository data revealed enhanced canonical Notch activation with upregulation of DLL1 and downregulation of *NOTCH4*.

**Conclusion:**

Our findings suggest that scrub typhus patients are characterized by enhanced canonical Notch activation. Elevated plasma levels of DLL1 were associated with organ dysfunction and poor outcomes in these patients.

## Introduction

Scrub typhus, a multi-system infection caused by a vector-borne obligate intracellular Gram-negative bacterium *Orientia tsutsugamushi* (*O. tsutsugamushi*), is endemic in the Asia–Pacific region [1]. It is manifested by fever and multiple organ involvement with significant mortality if untreated [2]. Despite causing an estimated 1 million cases per year and an increasing global presence, mechanisms of scrub typhus pathogenesis remain unclear [3]. Scrub typhus is characterized by widespread vasculitis and perivasculitis, accompanied by infiltration of mononuclear cells into affected organs [4]. Endothelial cells and cells involved in innate immunity, such as dendritic cells and macrophages, are the primary targets of *O. tsutsugamushi* infection in vivo. Macrophages secrete multiple inflammatory cytokines and chemokines upon *O. tsutsugamushi* infection but fail to control bacterial growth at the initial infection site as well as in inflamed target organs [5, 6]. Thus, the intracellular pathogen, *O. tsutsugamushi*, actively evades cellular autophagy, required not only for direct elimination of cytosolic pathogens, but also for processing and presentation of intracellular antigens to surrounding T cells [7]. These pathogenic phenomena, including acute CD4 + T lymphopenia by massive apoptosis and activation of monocytes/macrophages and endothelial cells, could be linked to the pathologic features observed in scrub typhus patients [8, 9].

Mammals possess four receptors (Notch 1–4) that are bound by five ligands of the Jagged family and Delta-like family (Jagged 1 and Jagged 2, and Delta-like ligand 1 (DLL1), DLL3 and DLL4) [10]. The Notch family molecules play a key role in a variety of developmental processes by controlling cell fate decisions and the Notch pathways are operating in a large number of biological processes in several organ systems, such as hematopoiesis, somatogenesis, vasculogenesis, neurogenesis, and homeostasis [10, 11]. The Notch family may also play an essential role in innate and adaptive immunity [10]. Notch signaling regulates T cell development, differentiation, and the functional responses of mature T cells as well as innate immunity cells [12]. Indeed, a role of Notch signaling in innate immune cells during non-sterile [13] and sterile inflammation such as cardiovascular disorders [14] has also recently been reported.

Several studies implement Notch signaling in the pathogenesis of various infections including regulation of the immune response to *Mycobacterium tuberculosis* [15] and promoting IFN-γ secretion during *Leishmania* infection [16]. A critical role of DLL1 was demonstrated in the pathogenesis of influenza A virus (H1N1) infection [17]. Although most reports indicate an inflammatory role of Notch in these infections, Notch signaling may also advance anti-inflammatory effects by promoting intracellular survival through downregulation of Toll-like receptors (TLR)2- and TLR4 [18].

Notch signaling has also been showed to influence the function of monocytes/macrophages and endothelial cells [10, 19, 20], and based on the importance of these cells in *O. tsutsugamushi* infection, we hypothesized a role for Notch signaling in scrub typhus infection. To the best of our knowledge, however, Notch signaling has not been investigated in scrub typhus patients. We hypothesized that Notch signaling could play an important role in regulating inflammation and immune activation as well as promoting organ dysfunction and mortality in these patients. To explore this, we measured plasma levels of DLL1 and the expression of Notch receptors in whole blood of scrub typhus patients. There are some similarities but also differences between DLL1 and DLL4, but although there are a few reports on plasma levels of DDL4 in cancer patients [21], based on our experience, DDL1 is the only Notch ligand that can be easily measured in serum/plasma. Finally, the regulation of Notch pathways by *O. tsutsugamushi* was assessed in human monocytes in microarray experiments [6] available in public repositories (see methods for link).

## Materials and methods

### Patients and controls

Patients > 15 years of age admitted to Christian Medical College, Vellore, Tamil Nadu, India between November 2009 and February 2011 with suspected scrub typhus were considered for inclusion into the study. Patients were considered to have scrub typhus if they had (i) a typical clinical manifestation scrub typhus and (ii) confirmed diagnosis of *O. tsutsugamushi* based on a positive IgM ELISA test (see below). In total, 129 patients were included as scrub typhus cases. The scrub typhus patients were further divided into subgroups according to disease severity. Those with no organ dysfunction were considered to have mild disease, those with one organ dysfunction moderate, while two or more organ dysfunctions were defined as severe disease. Organ dysfunction was defined as follows: renal dysfunction: creatinine ≥ 2.5 mg/dl; hepatic dysfunction: bilirubin (total) ≥ 2.5 mg/dl; pulmonary dysfunction: bilateral pulmonary shadows on chest X-rays with moderate or severe hypoxia (SpO_2_ < 90%); cardiovascular dysfunction: systolic blood pressure < 80 mmHg despite fluid resuscitation; and central nervous system dysfunction: significant altered sensorium with Glasgow Coma Scale (GCS) ≤ 8/15. The patients confirmed to have scrub typhus were treated with doxycycline with or without azithromycin. Treatment including mechanical ventilation and vasoactive agents was decided by the treating physician as per protocol. All patients were followed-up until discharge from the hospital or in-hospital death.

Two control groups were included. One group was patients admitted with acute febrile illness but confirmed to have an alternate infection with negative scrub typhus ELISA. Of these patients, six had dengue fever, four typhoid fever, three influenza, two tuberculosis, two acute encephalitis, one aseptic meningitis, one leptospirosis, one pneumonia, one liver abscess, one urosepsis, one rubella, one viral hepatitis, and seven had infectious disorders of uncertain etiology. In addition, 31 healthy controls (14 female, 17 male) were included. The criteria for the section of the healthy control group were: (i) apparently healthy based on self-reported disease history, (ii) living in the same area of South India as the patients, and (iii) being able to deliver blood samples in the same time period as the patients.

### Blood sampling procedure

Blood samples were collected at first presentation, before specific treatment, and a follow-up sample was obtained 1–2 weeks after the initial sample, i.e., after treated with doxycycline with or without azithromycin. Peripheral venous blood was drawn into pyrogen-free, vacuum blood collection tubes with EDTA as anticoagulant (4 × 7.5 ml), centrifuged within 30 min at 2000 × *g* for 20 min to obtain platelet-poor plasma that was stored in multiple aliquots at − 80 °C until analysis. All samples were thawed no more than three times.

For RNA analyses, whole blood were collected in BD PAXgene™ Blood RNA tubes (10 ml) using MagMAX™ for Stabilized Blood Tubes RNA Isolation Kit (Invitrogen™, Waltham, MA) following the manufacturer’s instructions.

### Microbiological diagnosis

Scrub typhus IgM ELISA was performed on serum samples using the Scrub Typhus Detect (InBios International, Inc., Seattle, WA). The determination of a cut-off value of 0.5 took place several years ago, relying on both the receiver operating characteristic curve and mean values from healthy volunteer samples, with + 3 standard deviations used to generate a region-specific cut-off OD value of 0.5. Further validation was done using known scrub typhus sera (confirmed by PCR/immunofluorescence) and sera from patients with other diseases like malaria and enteric fever and healthy controls. We also used a positive and a negative control provided in the kit as well as an in-house positive control for every run. This test has a sensitivity and specificity of > 90% [22]. The IgM test has recently been further described by Jain et al. [23].

### Enzyme immunoassays (EIAs)

Plasma levels of DLL1, C-reactive protein (CRP), soluble (s) CD163, sCD14, macrophage migration inhibitory factor (MIF), YKL-40 (also known as Chitinase 3-like 1 [CHI3L1]), vascular cell adhesion molecule (VCAM)-1, interleukin (IL)-6, tumor necrosis factor (TNF), and IL-10 were measured by EIAs obtained from R&D Systems (Minneapolis, MN) [8]. The intra- and inter-assay coefficients of variations were < 10% for all EIAs. To further minimize run-to-run variability, serial samples from a given individual were analyzed on the same tray.

### *Real-time* PCR analyses of Notch receptors and DLL in whole blood

Total RNA was obtained with the use of RNeasy spin columns (QIAGEN, Hilden, Germany). All samples were subjected to DNase treatment (RNase-Free DNase Set; QIAGEN) and stored at − 80 °C until further analysis. cDNA synthesis was performed using the High-Capacity cDNA Reverse Transcriptation Kit (Applied Biosystems, Foster City, CA). Gene expression was examined by real-time quantitative PCR, and mRNA level of *NOTCH1, NOTCH2,* and *NOTCH4* and reference genes *GAPDH* and *ACTB* was assessed with SybrGreen PCR kit (Applied Biosystems). Sequence-specific intron spanning oligonucleotide primers were designed by NCBI Primer Blast. The relative mRNA level of each transcript was calculated by a relative standard curve and normalized to the reference genes. Primer sequences *NOTCH1* (forward primer 5´-TGGTCAGGGAAATCGTGCCA-3′, reverse primer 5′-GGTAGGAGCCGACCTCGTT-3´), *NOTCH2* (forward primer 5′- TGACTGCCTTGCCAATCCTT-3′, reverse primer 5′- CTCATTCATGTCTGTCTGGCACTT-3′), *NOTCH4* (forward primer 5′- TCTCCGGCACCCGATGT-3′, reverse primer 5′- TCAAAGCCTGGGAGACACTTG-3′), *GAPDH* (forward primer 5′- CCAAGGTCATCCATGACAACTT-3′, reverse primer 5′- AGGGGCCATCCACAGTCTT-3′), ACTB (forward primer 5′- AGGCACCAGGGCGTGAT-3′, reverse primer 5′- TCGTCCCAGTTGGTGACGAT-3′).

### Evaluation of DLL1 and Notch receptor expression from public databases

mRNA expression from relevant studies of *O. tsutsugamushi stimulation *in vitro in monocytes in microarray experiments available in public repositories (https://www.ncbi.nlm.nih.gov/geo/query/acc.cgi?acc=GSE24247).

### Statistics

Differences in plasma DLL-1 and mRNA expression of Notch receptors between patients with scrub typhus, acute infection controls, and healthy controls were compared with the Kruskal–Wallis test followed by Dunn’s post hoc test. Paired differences (i.e., within scrub typhus group) were compared using the Wilcoxon signed-rank test. The association between plasma DLL1 and mRNA expression of Notch receptors and disease severity, organ dysfunction, and outcome was evaluated by the Mann–Whitney U-test. The association between DLL1 and mortality (*n* = 7) was further investigated by receiver operation curve (ROC) analysis and as logistic regression using log10 DLL1/SD as predictor and one-by-one adjustment with relevant covariates. The correlation between DLL1, mRNA expression of Notch receptors and inflammatory markers was evaluated using Spearman correlation and was assessed within the groups of scrub typhus, acute infection controls, and healthy controls. *p* values are two-sided and considered significant when < 0.05.

## Results

### Plasma levels of DLL1 and expression of Notch receptor in whole blood at admission and during recovery in patients with scrub typhus

**Fig. 1:**
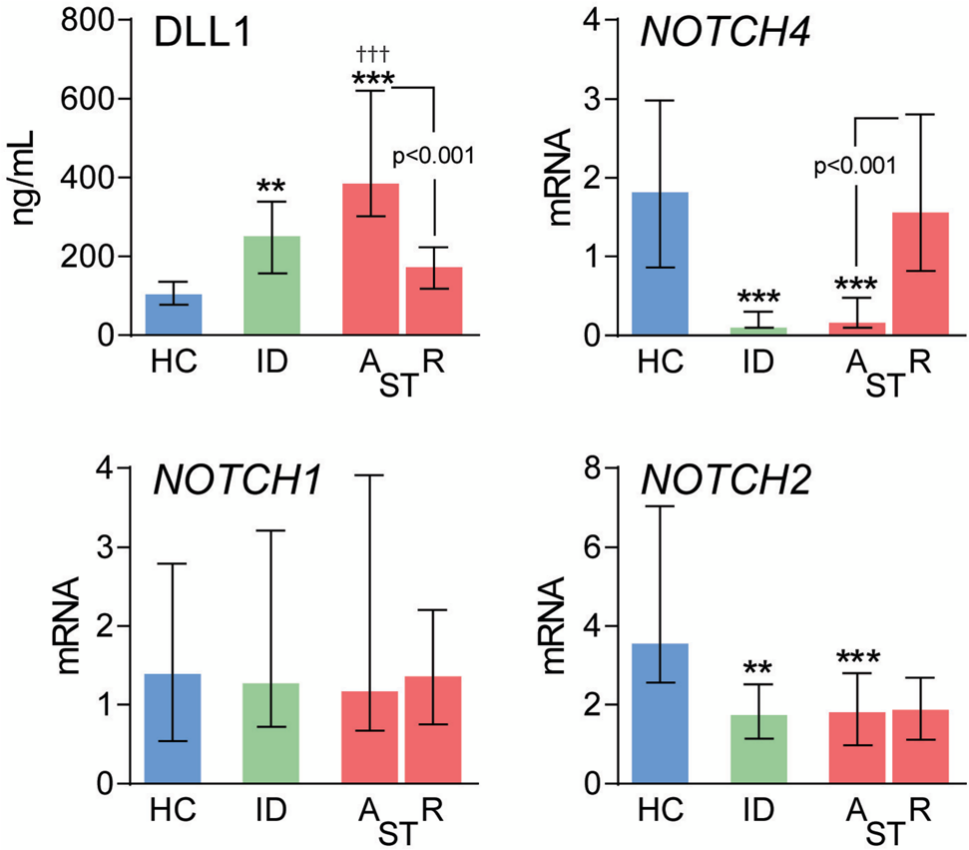
Levels of DLL1 and *NOTCH* receptors in scrub typhus patients and controls. Plasma levels of Delta-like canonical Notch ligand 1 (DLL1) and mRNA levels of *NOTCH*-receptors 1, 2, and 4 in peripheral blood in scrub typhus (ST) patients (*n* = 129) on admission (**A**) and at recovery (*R*), 1–2 weeks after the initial sample, i.e., after treated with doxycycline with or without azithromycin, as well as comparative levels in infectious disease controls (ID, *n* = 31) and healthy controls (HC, *n* = 31). Data are given as medians and interquartile range (IQR) and were compared between groups with Kruskal–Wallis test followed by Dunn’s post hoc test. *p < 0.05, **p < 0.01 and ***p < 0.001 versus healthy controls. ^†††^*p* < 0.001 versus ID controls. Comparisons between levels at admission and recovery were compared using the Wilcoxon signed-rank test and is shown with p value

Plasma levels of the Notch ligand DLL1 were analyzed in patients with scrub typhus (*n* = 129), febrile infectious disease controls (*n* = 31) (Table 1), and healthy controls (*n* = 31). Levels of DLL1 were markedly higher in scrub typhus patients at admission compared to healthy controls and infectious controls (Fig. 1). In recovering scrub typhus patients, there was a significant decrease in DLL1 levels compared to admission levels.

**Table 1:** Characteristics of patients with scrub typhus according to disease severity and infectious disease controls

	Infectious controls (*n* = 31)	Mild disease (*n* = 51)	Moderate disease (*n* = 37)	Severe disease (*n* = 41)
Age (mean ± SD)	35 ± 18	50 ± 14	45 ± 17	40 ± 16
Gender (male/female)	17/14	24/27	25/12	17/24
Renal dysfunction, *n* (%)	2 (7)	0	6 (16)	15 (37)
Hepatic dysfunction, *n* (%)	4 (13)	0	7 (19)	24 (59)
CNS dysfunction, *n* (%)	1 (3.2)	0	0	1 (2.4)
Respiratory dysfunction, *n* (%)	1 (3)	0	10 (27)	26 (63)
Circulatory dysfunction, *n* (%)	4 (13)	0	5 (14)	20 (49)
Creatinine, mg/dL	1.2 (0.9, 1.4)	1.2 (1.0, 1.7)	1.2 (1.0, 1.7)	1.8 (1.1, 3.3)
eGFR	74 (59, 85)	60 (46, 81)	65 (38, 80)	36 (19, 72)
Total bilirubin, mg/dL	0.8 (0.4, 1.6)	0.7 (0.5, 1.0)	1.4 (0.9, 2.1)	2.8 (1.3, 6.0)
Total s-protein,	6.6 (6.3, 7.1)	6.8 (6.3, 7.4)	6.4 (6.0, 7.1)	6.1 (5.8, 6.7)
Albumin, g/dL	3.4 (3.0, 3.8)	3. 0 (2.6, 3.3)	2.7(2.4, 3.0)	2.6 (2.4, 2.7)
AST, U/L	55 (46, 145)	123 (84, 175)	143 (96, 201)	190 (112, 247)
ALT, U/L	36 (20, 93)	65 (45, 121)	84 (48, 109)	65 (52, 117)
Alkaline phosphatase, U/L	112 (74, 182)	122 (85, 176)	158 (120, 230)	219 (170, 306)

Data for the biochemical parameters in serum are given as medians and interquartile range (*IQR*), *AST* aspartate aminotransferase; *ALT* alanine aminotransferase; *eGFR* estimated glomerulus filtration rate

When examining the mRNA levels in whole blood of the corresponding Notch receptors, we found an opposite pattern, with markedly decreased expression of *NOTCH4*, and a more modest decreased *NOTCH2* expression, at admission in both scrub typhus patients and in infectious controls comparing healthy controls (Fig. 1). During recovery, *NOTCH4* expression in scrub typhus patients increased to the levels in healthy controls while the expression of *NOTCH2* remained suppressed. Expression of *NOTCH1* was not regulated in scrub typhus patients or in infectious disease controls (Fig. 1).

### Plasma levels of DLL1 and expression of Notch receptors in scrub typhus patients in relation to disease severity, organ dysfunction, and mortality

**Fig. 2:**
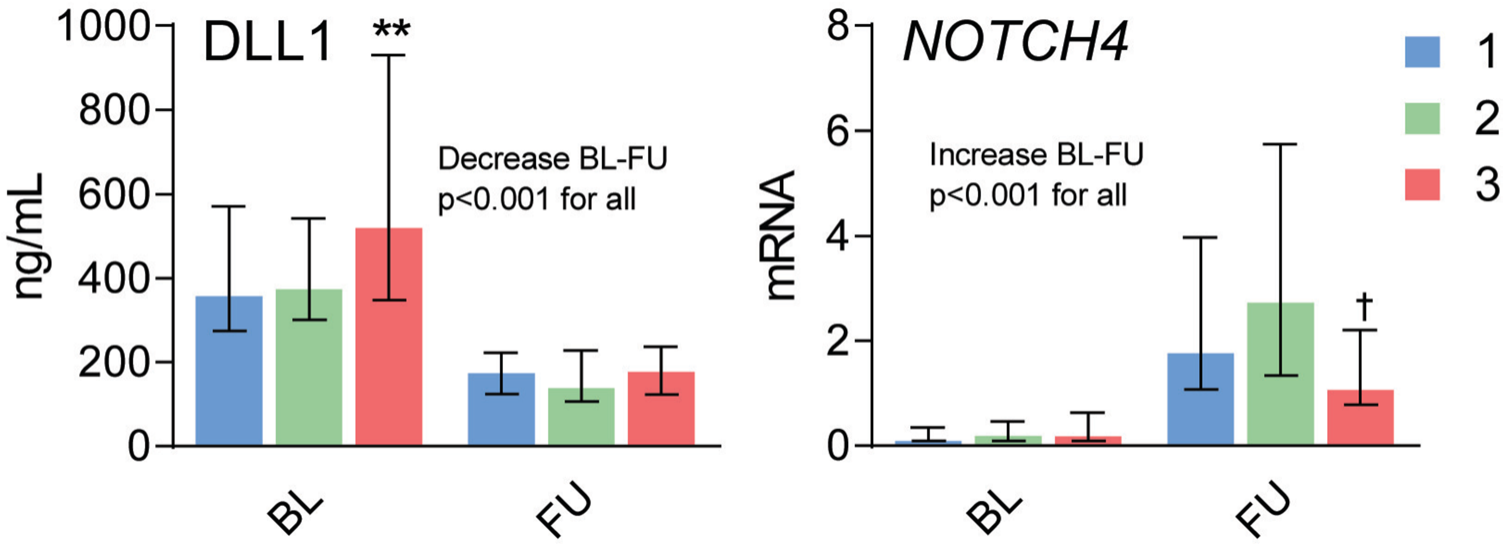
Levels of DLL1 and *NOTCH4* in relation to disease severity of scrub typhus patients. Plasma levels of Delta-like canonical Notch ligand 1 (DLL1) and mRNA levels of *NOTCH4* in peripheral blood in scrub typhus patients (*n* = 129) on admission (baseline, BL) and at recovery (follow-up, FU, 1–2 weeks after the initial sample, i.e., after treated with doxycycline with or without azithromycin) in relation to disease severity where patients with no organ dysfunction were considered to have mild disease (*n* = 51, blue bars), those with one organ dysfunction moderate disease (*n* = 37, green bars), while two or more organ dysfunctions were defined as severe disease (*n* = 41, red bars). Data are given as medians and interquartile range (IQR) and were compared between groups with Kruskal–Wallis test followed by Dunn’s post hoc test. **p < 0.01 versus mild disease; ^†^*p* < 0.05 versus moderate disease. Comparisons between levels at admission and recovery were compared using the Wilcoxon signed-rank test and is shown with p value

All patients were followed-up until discharge from the hospital or in-hospital death. During a median follow-up of 27 days (range 6–137 days), 78 patients with *O. tsutsugamushi* infection developed organ dysfunction and 7 patients died (Table 1). Patients with scrub typhus were classified in relation to disease severity as mild disease (*n* = 51, no organ dysfunction), moderate disease (*n* = 37, one organ dysfunction), and severe disease (*n* = 41, two or more organ dysfunction) (Table 1). At baseline, but not at follow-up, high plasma DLL1 at admission was associated with disease severity with significantly higher levels in those with severe scrub typhus comparing those with mild disease (Fig. 2). Further analysis revealed that DLL1 was associated with renal and circulatory dysfunction, but not with hepatic and respiratory dysfunction (Table 2).

**Table 2:** Plasma DLL1 and expression of Notch receptors in relation to severity, organ dysfunction, and outcome

	sDLL-1	*NOTCH1*	*NOTCH2*	*NOTCH4*
Severity, 1/2 *versus* 3, r* (*p*)	1.40 (0.001)	1.01 (0.95)	0.99 (0.91)	1.20 (0.12)
Renal dysfunction, r* (*p*)	1.82 (< 0.001)	0.97 (0.83)	1.04 (0.76)	1.33 (0.024)
Hepatic dysfunction, r* (*p*)	1.22 (0.076)	0.91 (0.50)	1.05 (0.69)	1.09 (0.49)
Respiratory dysfunction, r* (*p*) (p)	0.97 (0.80)	1.24 (0.083)	0.98 (0.86)	1.24 (0.074)
Circulatory dysfunction, r* (*p*)	1.56 (< 0.001)	1.12 (0.39)	0.98 (0.91)	1.09 (0.51)
Death, r* (p)	1.84 (< 0.001)	1.03 (0.90)	0.76 (0.29)	1.19 (0.39)

^*^ratio of rank from Mann–Whitney U-test between patients with and without severity or outcome. *DLL1* delta-like canonical Notch ligand 1

In contrast, *NOTCH4* expression was markedly suppressed in all disease severities at admission, but although all subgroups of patients showed an increase in *NOTCH4* mRNA expression during follow-up, those with severe disease had significantly lower expression than moderate disease at recovery (Fig. 2). *NOTCH4* expression (at follow-up) was only associated with renal dysfunction in these patients (Table 2). In contrast, *NOTCH2* and *NOTCH1* expression showed no relation to disease severity (data not shown).

**Fig. 3:**
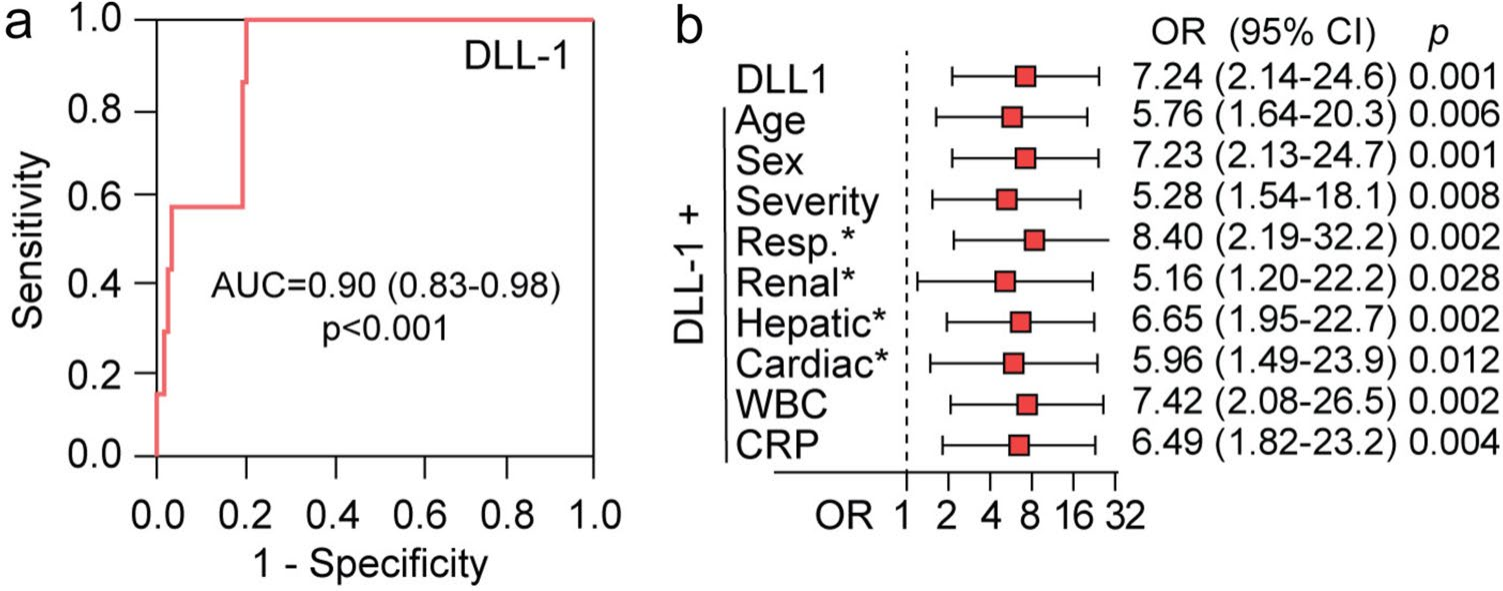
Plasma DLL1 and survival in scrub typhus patients. **A** Receiver operating characteristic (ROC) analysis showing associations between mortality and DLL1 levels in scrub typhus patient on admission. AUC and standard error are given with corresponding p value in parenthesis. **B** Logistic regression showing admission DLL1 (log10 DLL1/SD) as a predictor of mortality unadjusted and following one-by-one adjustment with relevant risk covariates. *organ dysfunction as defined in methods. *DLL1* delta-like canonical Notch ligand 1

Finally, DLL1 plasma levels were associated with a fatal outcome in scrub typhus patients (Table 2), and ROC analyses revealed that high DLL1 appeared to be a strong marker for short-term survival (AUC of 0.90, *p* < 0.001) in these patients (Fig. 3). Furthermore, in logistic regression (Fig. 3B), a 1-SD increase in plasma DLL1 was associated with a ~ 7 times higher risk of death. This association was not markedly attenuated when adjusted one-by-one with relevant covariates associated with poor prognosis such as severity, organ dysfunction, or acute-phase inflammation as reflected by CRP.

### Plasma levels of DLL1 and Notch receptors expression in relation to markers of inflammation and endothelial cell activation

We have previously shown that *O. tsutsugamushi* infection is associated with markers of monocyte/macrophage and endothelial cell activation [8]. As shown in Table 3, admission DLL1 levels in scrub typhus patients were robustly positively correlated with general inflammation as reflected by CRP and its inducer IL-6 and monocyte/macrophage activation as reflected by sCD163 sCD14, MIF, TNF, and IL-10 as well as markers of vascular inflammation as reflected by YKL-40 and VCAM-1. In infectious disease controls, a similar pattern was observed, but without correlations between DLL1 *and* MIF and IL-10. In contrast, Notch receptor expression was not correlated with monocyte/macrophage or vascular activation markers in scrub typhus.

**Table 3:** Plasma levels of DLL1 and expression of Notch receptors in relation to markers of inflammation, immune activation, and vascular inflammation in healthy controls (HC), infectious controls (IC), and patients with scrub typhus (ST)

	sDLL1			*NOTCH1*			*NOTCH2*			*NOTCH4*		
Grp:	HC	IC	ST	HC	IC	ST	HC	IC	ST	HC	IC	ST
CRP	0.20	0.42*	0.34**	0.19	− 0.13	0.02	0.01	0.12	0.01	0.02	0.10	0.04
CD163	0.18	0.61**	0.58**	–0.15	− 0.36	–0.06	–0.03	0.17	0.11	0.02	–0.46	0.03
sCD14	− 0.14	0.56**	0.41**	–0.34	–0.67**	–0.09	–0.29	–0.17	0.13	–0.18	–0.26	–0.09
MIF	0.37*	− 0.11	0.41**	–0.10	0.06	–0.05	–0.25	0.06	–0.06	–0.10	–0.10	0.08
YKL40	0.33	0.39*	0.53**	0.12	− 0.32	0.11	–0.29	–0.07	0.06	0.14	–0.31	0.17
VCAM1	0.16	0.48*	0.51**	–0.34	− 0.31	–0.10	–0.27	0.16	0.07	–0.08	–0.15	–0.09
IL-6	− 0.34	0.35	0.38**	–0.22	0.01	0.11	–0.02	0.15	0.00	–0.21	0.00	0.05
TNF	0.41*	0.68**	0.64**	–0.19	− 0.39	0.09	–0.17	0.20	0.03	–0.22	–0.15	0.03
IL-10	0.12	0.16	0.49**	0.05	− 0.39	0.08	0.20	0.19	0.04	–0.19	–0.29	0.07

Data are given as Spearman’s rank correlation coefficient (rho) and * represents *p* value: *p < 0.05, **p < 0.001

### Regulation of Notch pathways in monocytes by *O. tsutsugamushi*

**Fig. 4:**
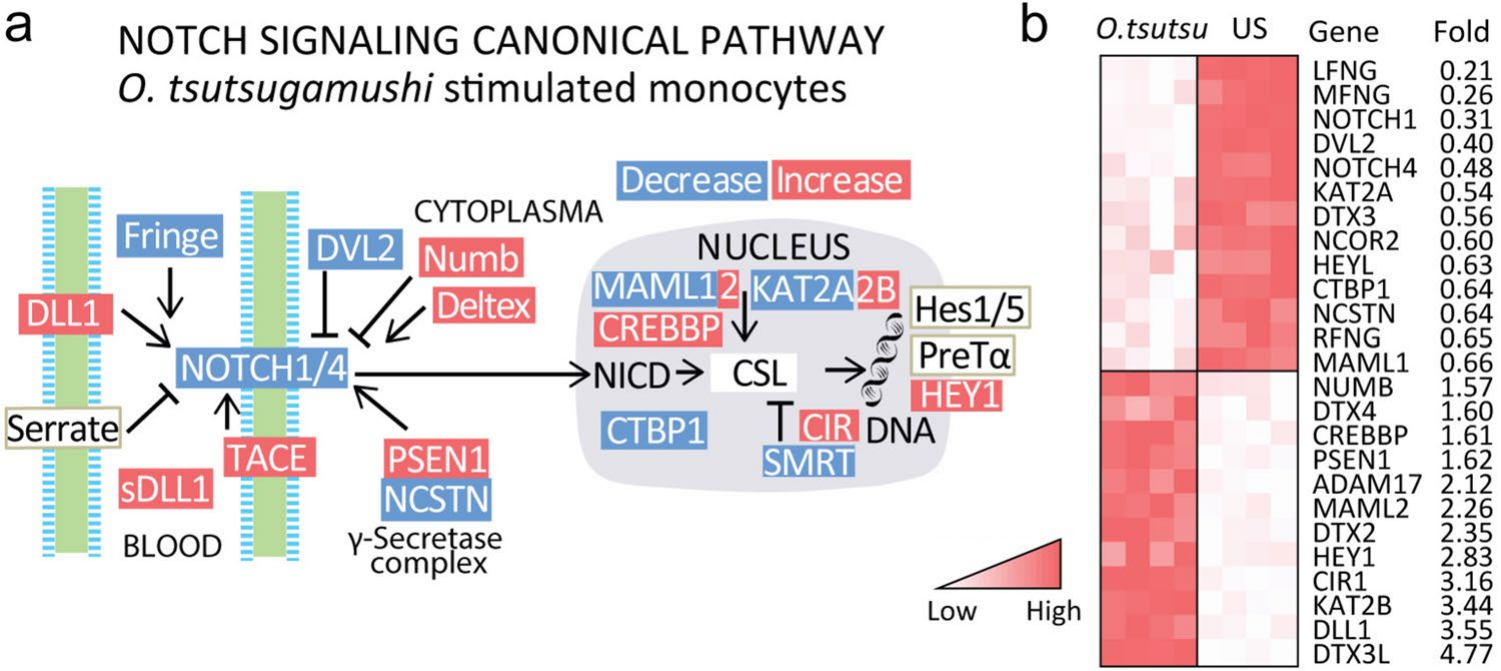
Regulation of Notch pathways by *O. tsutsugamushi* in mononuclear cells. The figure summarizes relevant genes for Notch canonical signaling in monocytes infected with *O. tsutsugamushi* compared to non-infected cells (MC, *n* = 4 in each group) from public repositories presented in the KEGG database (**A**) and as a heatmap (**B**) over the regulated mRNAs. Significant results in Fig. 4A (FDR adjusted p values) are indicated in red/blue (for increased/decreased mRNA expression) (**A**) and with red indicating a higher expression (**B**)

We finally probed regulation of Notch pathways by *O. tsutsugamushi *in vitro in monocytes in microarray experiments available in public repositories [5, 6]. Figure 4A shows regulation of the Notch pathways as outlined by KEGG (Kyoto encyclopedia of genes and genomes) in monocytes infected with *O. tsutsugamushi*. The overall impression is an enhanced canonical Notch activation in *O. tsutsugamushi*-infected monocytes. Thus, in line with our in vivo data in scrub typhus patients, these experiments show a marked upregulation of *DLL1* and a reciprocal downregulation of *NOTCH4*. Moreover, they show prominent downstream readout of Notch signaling such as *CREBBP* and *HEY1*, both identified as a specific molecular target for Notch signaling. These data are also shown as a heatmap in Fig. 4B, illustrating fold change in regulated Notch-related mRNAs in monocytes infected with *O. tsutsugamushi* compared to non-infected cells.

## Discussion

Our study shows that scrub typhus patients have elevated plasma DLL1 and downregulated gene expression of the receptor *NOTCH4* in whole blood, with normalization of both during follow-up. Notably, plasma DLL1 in scrub typhus patients was associated with disease severity and short-term mortality. Further, DLL1 robustly correlated with elevated levels of markers of monocyte/macrophages and vascular inflammation [8]. Finally, regulation of Notch pathways in monocytes infected with *O. tsutsugamushi*, as evaluated by public gene expression repositories, revealed an overall impression of an enhanced canonical Notch activation with upregulation of *DLL1* and a reciprocal downregulation of *NOTCH4* supporting our in vivo observations.

Several studies suggest a complex role of Notch signaling in various bacterial infections (e.g., *Mycobacterium tuberculosis, Mycobacterium leprae, and Helicobacter pylori*), viral infections (e.g., *influenza virus infection, hepatitis B virus, and hepatitis C virus* infection), fungal infections (e.g., *Cryptococcus and histoplasmosis infection*), and parasite infection (e.g., *Leishmania major* infection) [13].To the best of our knowledge, this is the first study to demonstrate involvement of Notch signaling in *O. tsutsugamushi* infection. Moreover, while experimental studies demonstrate that dysregulated Notch signaling may promote immune response and inflammation in various infections [13], fewer studies have evaluated dysregulation of these pathways in clinical samples. Our finding of increased Notch ligand DLL1 in plasma and decreased expression of the receptor Notch4 whole blood in scrub typhus patients was mirrored in vitro in monocytes infected with *O. tsutsugamushi*. Although less dysregulated, a similar pattern was seen in the heterogeneous group of febrile patients with various microbiological etiology. Thus, we do not suggest dysregulated Notch signaling is specific for *O. tsutsugamushi* infection, but like other mediators of inflammation and immune activation, Notch signaling may play a pathogenic role in various disorders and the present study suggest that this also includes scrub typhus.

Notch signaling is involved in both adaptive and innate immunity and focus has been on the regulation in various T cell subsets [24]. However, several studies have demonstrated involvement of Notch signaling in monocytes/macrophages and endothelial cells, cell types that are of major importance in the pathogenesis of *O. tsutsugamushi* infection. Thus, Notch signaling seems to be involved in polarization to the inflammatory M1 macrophage subtype [25] and may modulate TLR signaling in monocyte/macrophages and interfere with mitochondrial function and oxidative stress in these cells [20]. Furthermore, a role in angiogenesis, vascular stability, and endothelial cell activation, with the potential to induce both apoptosis and proliferation of these cells, has also been demonstrated [26]. In our study, DLL1 levels correlated with markers of monocyte/macrophage and endothelial cell activation, markers we previously have shown to associate with adverse outcome in these patients [8]. Moreover, *DLL1* was upregulated and *NOTCH4* was downregulated by *O. tsutsugamushi*-infected monocytes in vitro. It is tempting to hypothesize that the association of DLL1 with adverse outcome in scrub typhus patients may involve a role in promotion of macrophage inflammation and endothelial dysfunction with vascular leakage, two hallmark of severe *O. tsutsugamushi* infection [27].

DLL1 may activate cellular immune responses promoting release of inflammatory cytokines [28]. During sepsis, DLL1 enhances endothelial dysfunction and vascular leakage [29]. Thus, the association of DLL1 with adverse outcome in scrub typhus patients could suggest DLL1 is not only a marker, but also a mediator of disease severity in these patients. In contrast to DLL1, *NOTCH4* was downregulated in whole blood in scrub typhus patients. Interestingly, Notch4 may inhibit endothelial cell apoptosis [30] and impair endothelial cell activation [31]. Moreover, in contrast to DLL1, Notch4 seems to promote an anti-inflammatory phenotype in activated macrophages [32]. Thus, high levels of DLL1 and low levels of *NOTCH4*, as seen in the present study, could potentially contribute to an inflammatory phenotype in scrub typhus patients. A similar pattern was seen in vitro in *O. tsutsugamushi-*exposed monocytes, with evidence for activation of canonical Notch signaling, further supporting such a notion.

The present study has some limitations such as a low number of patients with fatal events. Moreover, only selected ligands/receptors in the Notch signaling pathways were evaluated in vivo and we lack data at the site of infection. Furthermore, *NOTCH* receptor expression was analyzed in whole blood, and the lack of correlation of these receptors and markers of monocyte/macrophage activation could indicate that the expression in whole blood reflects levels in several cell populations in addition to monocytes. Furthermore, we lack data on protein expression of these receptors. Moreover, several correlations were performed and some of the findings could be by chance. Also, correlations do not necessarily imply causal relationship.

Nonetheless, this first report of Notch signaling in scrub typhus patients suggesting that an imbalance between enhanced DLL1 levels and decreased *NOTCH4* expression could contribute to the pathogenesis of *O. tsutsugamushi* infection.
